# Rotation-Free Scalar Calibration of Cubic Magnetic Gradient Tensor Array Using Constant-Magnitude Magnetic Fields with Randomized Orientations

**DOI:** 10.3390/s26082521

**Published:** 2026-04-19

**Authors:** Chen Wang, Ziqiang Yuan, Gaigai Liu, Yingzi Zhang, Wenyi Liu

**Affiliations:** State Key Laboratory of Extreme Environment Optoelectronic Dynamic Measurement Technology and Instrument, North University of China, Taiyuan 030051, China; b20220620@st.nuc.edu.cn (C.W.); s202506112@st.nuc.edu.cn (Z.Y.);

**Keywords:** magnetic gradient tensor (MGT), fluxgate magnetometer array, constant-magnitude magnetic field, Helmholtz coil system, pseudo-gradient suppression

## Abstract

Accurate calibration is essential for ensuring the performance of magnetic gradient tensor (MGT) arrays. Existing calibration methods generally rely on mechanical rotation to obtain magnetic responses under multiple orientations. However, for large-scale cubic MGT arrays, rotating the entire array using a high-precision non-magnetic turntable is often costly and impractical, while manual rotation is difficult to control and may introduce array-center offsets. To address these limitations, this paper proposes a rotation-free scalar calibration framework for cubic MGT arrays, in which a tri-axial Helmholtz coil system generates constant-magnitude magnetic fields with randomized orientations while compensating for ambient magnetic drifts. Based on the acquired data, a hierarchical calibration algorithm is developed to estimate sensor-level intrinsic errors and array-level misalignment errors. Experimental results show that the proposed method reduces the joint tensor invariant $C_{T}$ from $9.07\times 10^{3}$ nT/m to 11.51 nT/m, corresponding to a 99.87% reduction. In addition, compared with a conventional rotation-based fast calibration method, the proposed framework further decreases the mean and RMS of the joint $C_{T}$ by 62.7% and 63.1%, respectively. These results demonstrate that the proposed framework improves the spatial consistency of the MGT array and provides a practical calibration solution for large-scale MGT array systems.

## 1. Introduction

Magnetic gradient tensor (MGT) measurement arrays, which utilize spatially distributed vector magnetometers to reconstruct the first-order spatial derivatives of magnetic fields, are indispensable for high-precision magnetic anomaly detection and target localization [1,2,3,4,5]. By providing richer multidimensional constraints than single-point measurements, MGT arrays significantly enhance complex applications such as geological surveying, unexploded ordnance (UXO) detection, underwater magnetic tracking, and space magnetometry [6,7,8,9]. In practice, these arrays are typically constructed using either superconducting quantum interference devices (SQUIDs) [10,11,12] or fluxgate magnetometers [13]. Although SQUID-based systems offer superior sensitivity, fluxgate magnetometers remain the preferred choice for portable and field-deployable MGT arrays due to their operational simplicity, cost-effectiveness, and structural flexibility.

However, the practical performance of distributed fluxgate MGT arrays is fundamentally bottlenecked by multi-source coupled distortions, making accurate calibration essential. Under field conditions, sensor-level intrinsic errors (e.g., scale-factor mismatch, non-orthogonality, and bias drift) intricately couple with array-level installation misalignments [14,15]. Since MGT array reconstruction relies on finite differencing across short spatial baselines, it is notoriously sensitive to minute measurement inconsistencies [16]. Consequently, these multi-source errors are dramatically amplified during differential calculation, generating severe pseudo-gradient artifacts. This is explicitly manifested in tensor invariants—such as the contraction tensor $C_{T}$ [17], which theoretically should be zero in a uniform field—exhibiting significant pseudo-fluctuations, and ultimately deteriorating the accuracy and reliability of target localization [18]. Therefore, accurate calibration is essential for ensuring the spatial consistency and localization reliability of MGT arrays.

From the perspective of optimization methodology, existing magnetometer calibration methods can be broadly grouped into deterministic model-based methods, stochastic optimization methods, and data-driven learning-based methods. Among them, conventional vector and scalar calibration methods predominantly belong to the deterministic model-based category, as both rely on explicit physical measurement models and structured parameter estimation under known field or scalar constraints [19,20,21]. Within this category, vector calibration relies on precisely known attitude references or externally generated standard fields, often requiring dedicated non-magnetic turntables and magnetic shielding rooms [14,22]. This makes it environmentally demanding, cost-prohibitive, and largely impractical for bulky MGT array structures. By contrast, scalar-based calibration [23,24] exploits the constant-magnitude property of the magnetic field to estimate parameters without requiring strict directional priors. Initiated by Merayo et al., who utilized an optically pumped magnetometer as a reference [25], scalar calibration algorithms have been subsequently optimized by Auster et al. [26], Foster and Elkaim [27], and Pang et al. [28,29]. These deterministic methods typically rely on least-squares, nonlinear least-squares, ellipsoid fitting, or coordinate transformation. They offer clear physical interpretability and high computational efficiency; however, their numerical conditioning often deteriorates significantly when applied to strongly coupled, high-dimensional array models. To circumvent these conditioning issues, stochastic optimization methods, such as particle swarm optimization and differential evolution, offer greater flexibility for nonconvex parameter estimation. Building on this, recent studies have continuously refined parameter estimation strategies: Li et al. [30] developed a novel calibration method using an improved Beluga Whale Optimization (BWO) algorithm to effectively increase population diversity and optimal parameter search capability, while Zhou et al. [31] introduced a hybrid framework combining Newton iteration and least squares to estimate the nine-parameter model and improve the signal-to-noise ratio. Recent years have also witnessed growing interest in machine learning and data-driven calibration methods for magnetic sensors. Existing studies in this direction can be broadly divided into two categories. The first category remains physics-informed, where neural networks are used to identify or optimize parameters of structured calibration models rather than replacing the models themselves. For example, Cowart and Petrich [32] embedded higher-order polynomial calibration models into a neural-network architecture, showing that such a framework can outperform classical hard-/soft-iron calibration while revealing an inherent tradeoff between calibration accuracy and model complexity. The second category is more purely data-driven, where the calibration model is learned directly from sensor input–output data. Alimi et al. [33] investigated an in situ AI calibration scheme for a three-axial magnetic sensor and showed that a neural-network-based approach can outperform conventional geometric calibration in low-power field deployment. More recently, Tian et al. [34] proposed an MLP-based data-driven calibration method for tri-axial magnetic sensors, which avoided explicit solving of error-model parameters and demonstrated improved static and dynamic calibration accuracy over traditional ellipsoid-fitting-based methods. These studies indicate that learning-based methods are promising for capturing nonlinear sensor distortions and coupling effects that are difficult to represent with low-order analytical models. Nevertheless, current machine-learning and data-driven calibration methods are still mainly developed for single tri-axial magnetic sensors rather than distributed MGT arrays, and their effectiveness typically depends on representative training data, controlled excitation conditions, or sufficiently uniform sampling coverage. Moreover, compared with deterministic physics-based calibration, their physical interpretability is generally weaker, and their transferability under changing environmental and array-level coupling conditions remains insufficiently established.

In addition to algorithmic methods, researchers have also extensively studied calibration from the perspective of hardware implementations and magnetic field excitation systems. For example, interference-compensated coil-based calibration systems have been developed to improve magnetometer calibration robustness under disturbed laboratory environments [35]. Automated estimation methods have been reported to identify sensor positions and orientations in magnetic measurement facilities by using well-characterized magnetic sources and optimization-based reconstruction [36]. In magnetic tracking systems, calibration-by-pivoting strategies have been proposed to simplify array calibration without requiring predefined poses of the magnetic target [37]. In addition, simultaneous calibration frameworks have been introduced for coupled magnetic sensing and actuation systems through large-scale joint optimization [38]. These studies have substantially enriched the methodological landscape of magnetic calibration. However, most of them are oriented toward single-sensor calibration, magnetic tracking/localization systems, or facility-level sensor-geometry correction, rather than cubic MGT arrays, whose performance critically depends not only on the accuracy of each individual triaxial sensor but also on the array-level spatial consistency required for finite-difference tensor reconstruction.

For spatially distributed vector magnetometer arrays, most of the above calibration frameworks—regardless of the specific optimization algorithm employed—still require mounting the entire structure on a turntable for manual or motorized multi-attitude manipulation in order to acquire multi-orientation sensor-response data [39]. Yet such dynamic mechanical rotation entails inherent limitations: it inevitably introduces motion-induced structural micro-deformations, centrifugal disturbances, and operator-dependent errors. More importantly, maintaining strict attitude synchronization across all distributed sensors during continuous rotation is practically infeasible. These rotation-induced synchronization errors are strongly coupled with array-level misalignments, fundamentally limiting the effectiveness of such methods in complex in situ environments. To eliminate the reliance on mechanical rotation, recent studies have explored active magnetic-field excitation and related system-level schemes for stationary in situ calibration of magnetic sensors [40,41]. In parallel, staged or decoupled calibration frameworks have also been proposed to separate sensor-level errors from array-level errors [42].

While these pioneering studies have laid a solid foundation for MGT array calibration, their practical deployment still faces several inherent constraints. First, traditional scalar calibration—whether relying on reference sensors or tensor invariants—typically overlooks slow variations in the background magnetic field. Consequently, the calibration performance is susceptible to environmental magnetic fluctuations, and the ultimate accuracy is constrained by the precision of the external reference sensors. Second, conventional vector calibration is heavily constrained by the reliance on dynamic mechanical rotations. Deploying bulky MGT arrays on non-magnetic turntables is highly impractical; moreover, this approach struggles to achieve comprehensive omnidirectional attitude scanning and inevitably introduces motion-induced errors and orientation alignment ambiguities. Finally, when existing in situ calibration methods are extended to a cubic MGT array (yielding 24-dimensional data), they typically treat parameter estimation as a monolithic global optimization problem. Moreover, existing scalar in situ methods often lead to a high-dimensional parameter estimation problem in which sensor-level distortions and array-level inconsistencies become difficult to distinguish under the weak constant-magnitude constraint, thereby degrading identifiability and numerical stability.

To address these challenges, this paper proposes a rotation-free, in situ scalar calibration framework for a cubic MGT array. The main contributions of this work are summarized as follows:

(1) We develop a rotation-free scalar excitation framework for cubic MGT arrays. By replacing mechanical rotation with controlled tri-axial Helmholtz-coil excitation, the proposed method generates constant-magnitude magnetic fields with randomized orientations while suppressing ambient magnetic drifts, thereby enabling stable multi-orientation calibration without moving the array.
(2) We propose a hierarchical calibration algorithm tailored for the cubic MGT array. Under approximately uniform excitation conditions, this method reparameterizes the complete multi-source error model into two processes: array-level affine consistency alignment and sensor-level absolute scalar normalization. Rather than attempting to directly estimate all physical error parameters in a single 24-channel global optimization, this method separates relative array-level errors from sensor-level bias and distortion at the correction algorithm level, thereby alleviating parameter coupling under constant-mode constraints and improving numerical robustness.
(3) We establish a comprehensive evaluation methodology utilizing six-face $C_{T}$ mapping and aggregated root-mean-square (RMS) invariants in uniform magnetic fields. This framework quantifies the suppression of pseudo-tensor artifacts and effectively verifies the restoration of spatial consistency across the high-dimensional MGT array.

## 2. System Description and Error-Theoretic Formulation

### 2.1. Cube Magnetic Gradient Tensor Array Geometry and Gradient-Tensor Reconstruction

**Figure 1:**
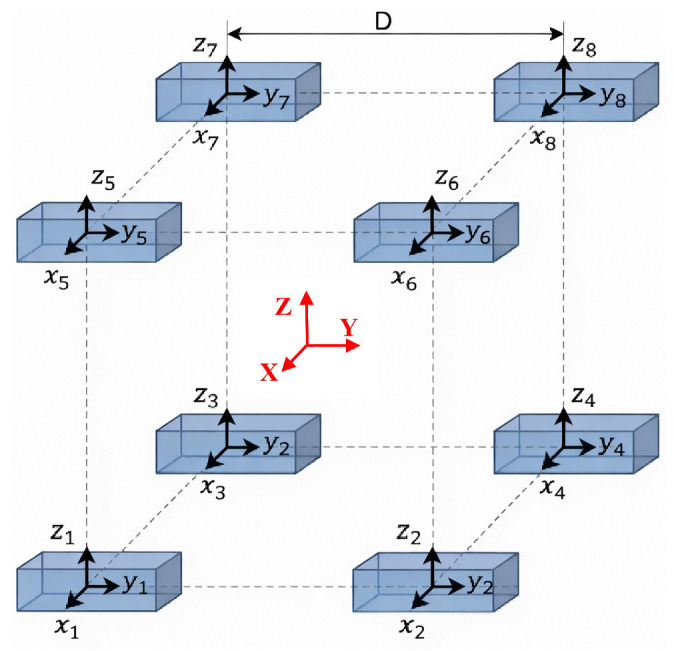
Cube Magnetic Gradient Tensor Array.

The Cubic Magnetic Gradient Tensor Array employed in this study consists of eight spatially distributed triaxial fluxgate sensors configured in a highly symmetrical cubic geometry. As illustrated in Figure 1, each sensor is mounted exactly at one of the eight vertices of the cube. To standardize the spatial alignment and subsequent mathematical modeling, the Array Reference Coordinate System (ARCS), denoted as $O\;-\;X_{a}Y_{a}Z_{a}$, is established. Its origin *O* is defined at the geometric center of the cubic array. The $X_{a}$, $Y_{a}$, and $Z_{a}$ axes are aligned parallel to the orthogonal edges of the cubic structure, with the spatial baseline lengths along the three coordinate axes denoted as $D_{x}$, $D_{y}$, and $D_{z}$, respectively.

The magnetic gradient tensor G is mathematically defined as the Jacobian matrix of the magnetic field vector $B={\left[B_{x},B_{y},B_{z}\right]}^{T}$ with respect to the spatial position coordinates $r={\left[x,y,z\right]}^{T}$:

(1) $$G=\left[\begin{array}{ccc} \frac{\partial B_{x}}{\partial x} & \frac{\partial B_{x}}{\partial y} & \frac{\partial B_{x}}{\partial z}\\ \frac{\partial B_{y}}{\partial x} & \frac{\partial B_{y}}{\partial y} & \frac{\partial B_{y}}{\partial z}\\ \frac{\partial B_{z}}{\partial x} & \frac{\partial B_{z}}{\partial y} & \frac{\partial B_{z}}{\partial z} \end{array}\right].$$

To facilitate the practical measurement of these gradient components, the cubic architecture allows each of its six faces to act as an independent planar measurement unit comprising four vertex sensors. The gradient tensor components at the center point of each face can be approximated using the spatial central finite-difference method along the two in-plane directions. Taking the $z^{-}$ face (where the outward normal is along the negative $Z_{a}$-axis, and the in-plane directions are $X_{a}$ and $Y_{a}$) as a representative example, the localized gradient tensor matrix at the center of this face can be formulated as:

(2) $$G_{z^{-}}=\left[\begin{array}{ccc} \frac{B_{x}^{1}+B_{x}^{2}-B_{x}^{3}-B_{x}^{4}}{D_{x}} & \frac{B_{x}^{2}+B_{x}^{3}-B_{x}^{1}-B_{x}^{4}}{D_{y}} & *\\ \frac{B_{y}^{1}+B_{y}^{2}-B_{y}^{3}-B_{y}^{4}}{D_{x}} & \frac{B_{y}^{2}+B_{y}^{3}-B_{y}^{1}-B_{y}^{4}}{D_{y}} & *\\ \frac{B_{z}^{1}+B_{z}^{2}-B_{z}^{3}-B_{z}^{4}}{D_{x}} & \frac{B_{z}^{2}+B_{z}^{3}-B_{z}^{1}-B_{z}^{4}}{D_{y}} & * \end{array}\right].$$

Here, $B_{i}^{j}$ (i∈{x,y,z},j=1,2,3,4) denotes the *i*-th component measured by the *j*-th triaxial fluxgate located at the four vertices of the $z^{-}$ face, and $D_{x}$ and $D_{y}$ are the corresponding in-plane baselines along *x* and *y*, respectively. In (2), the first two columns provide face-centered central finite-difference estimates of ∂B/∂x and ∂B/∂y at the face center and therefore retain the standard second-order truncation accuracy with respect to the in-plane baselines. The asterisks “*” are used only as a compact notation for the normal-derivative terms ∂B/∂z. In a quasi-static source-free region, these terms can be completed consistently using Maxwell constraints ∇·B=0 and ∇×B=0, e.g., $\partial B_{x}/\partial z=\partial B_{z}/\partial x$, $\partial B_{y}/\partial z=\partial B_{z}/\partial y$, and $\partial B_{z}/\partial z=-\left(\partial B_{x}/\partial x+\partial B_{y}/\partial y\right)$. The same construction applies to the other five faces by selecting the corresponding vertex sensors and baselines.

As formulated in the matrix above, the local magnetic gradient tensor is reconstructed via a first-order finite-difference scheme using vector-field samples acquired at the corner sensors. This algebraic approximation implicitly assumes homogeneous sensor responses and ideal orthogonal alignment of both sensing axes and array geometry. In practice, however, each triaxial fluxgate measurement is affected by sensor-intrinsic imperfections (e.g., scale-factor mismatch, axis non-orthogonality, and bias) as well as array-level installation and attitude misalignments, so that the field vectors at the vertices deviate systematically from the ideal samples. Since the tensor entries are obtained from linear combinations of these discrete measurements, even small single-sensor distortions are directly mapped into the finite-difference terms. Moreover, under the trace-free constraint commonly imposed in source-free magnetostatic reconstruction, errors in the in-plane components (e.g., $G_{xx}$ and $G_{yy}$) can further couple into the inferred out-of-plane term $G_{zz}$, amplifying the impact of residual mismatch. These considerations motivate a unified, multi-source error model that explicitly accounts for sensor- and array-level nonidealities in the subsequent formulation.

### 2.2. Multi-Source Error Model of the MGT Array

The accuracy of the reconstructed gradient tensor is intrinsically limited by the error propagation from individual discrete sensors. To mathematically quantify these distortions and decouple them from the true spatial magnetic field, a comprehensive multi-source error model must be established. For the cubic Magnetic Gradient Tensor Array, the measurement errors for the *i*-th sensor (i=1,2,…,8) primarily stem from two levels: the internal intrinsic distortions of the sensor itself and the external array-level installation misalignments.

Consider a cubic Magnetic Gradient Tensor Array (N=8) rigidly mounted on a cube. Let {ARCS} denote the array frame and $\left\{S_{n}\right\}$ the local sensor frame of the *n*-th magnetometer. The ideal magnetic field vector at sensor location $r_{n}$ expressed in {ARCS} is $B_{n}^{ARCS}=B\left(r_{n}\right)$. Each tri-axial sensor output is affected by axis-wise scale-factor mismatch, non-orthogonality, and additive bias. These deterministic effects are modeled by

(3) $$u_{n}=T_{n}\;B_{n}^{S_{n}}+b_{n}+\epsilon_{n},$$

where $u_{n}\in R^{3}$ is the measured field vector in sensor coordinates, $\epsilon_{n}$ is measurement noise, and 

(4) $$T_{n}≜K_{n}A_{n},\;K_{n}=\mathrm{diag}\left(k_{x,n},k_{y,n},k_{z,n}\right).$$

Here, $K_{n}$ captures directional sensitivity errors, $A_{n}$ captures the sensor-axis non-orthogonality (typically parameterized by three small angles), and $b_{n}={\left[b_{x,n},\;b_{y,n},\;b_{z,n}\right]}^{T}$ is the bias vector.

In a cubic Magnetic Gradient Tensor Array, gradient calculation strictly requires all sensors to be perfectly aligned with the Array Reference Coordinate System (ARCS, $O-X_{a}Y_{a}Z_{a}$). However, due to mechanical assembly tolerances, the local coordinate system of the *n*-th sensor possesses a spatial angular misalignment relative to the ARCS. This misalignment can be described by a 3×3 direction cosine rotation matrix $R_{n}$, characterized by three euler angles (pitch $\psi_{n}$, roll $\phi_{n}$, and yaw $\theta_{n}$). Therefore, the relationship between the true field in the ARCS ($B_{n}^{ARCS}$) and the true field projected in the *n*-th sensor’s local orthogonal frame ($B_{n}^{S_{n}}$) is:

(5) $$B_{n}^{S_{n}}=R_{n}B_{n}^{\mathrm{ARCS}},\;R_{n}=R_{z}\left(\theta_{n}\right)R_{y}\left(\phi_{n}\right)R_{x}\left(\psi_{n}\right),$$

where $\psi_{n},\phi_{n},\theta_{n}$ are the *X*–*Y*–*Z* euler angles.

Substituting the rotation relation into the intrinsic model yields the unified measurement equation

(6) $$u_{n}=T_{n}\;R_{n}\;B_{n}^{ARCS}+b_{n}+\epsilon_{n}.$$

For compactness, all calibration parameters of sensor *n* can be collected into

(7) $$\xi_{n}={\left[k_{x,n},\;k_{y,n},\;k_{z,n},\;\alpha_{n},\;\beta_{n},\;\gamma_{n},\;b_{x,n},\;b_{y,n},\;b_{z,n},\;\psi_{n},\;\phi_{n},\;\theta_{n}\right]}^{T},$$

where $\alpha_{n},\beta_{n},\gamma_{n}$ parameterize $A_{n}$. Stacking all sensors’ outputs as a 24-dimensional observation vector

(8) $$u≜{\left[u_{1}^{T},\;u_{2}^{T},\;\ldots ,\;u_{8}^{T}\right]}^{T}\in R^{24},\;B^{A}≜{\left[{\left(B_{1}^{A}\right)}^{T},\;\ldots ,\;{\left(B_{8}^{A}\right)}^{T}\right]}^{T}\in R^{24},$$

we obtain the array-level affine model

(9) $$u=\underset{H\left(\Xi \right)\in R^{24\times 24}}{\underset{︸}{\mathrm{blkdiag}(T_{1}R_{1},\ldots ,T_{8}R_{8})}}B^{A}+b_{\mathrm{arr}}+\epsilon ,$$

with $b_{\mathrm{arr}}={\left[b_{1},\ldots ,b_{8}\right]}^{T}$, $\epsilon ={\left[\epsilon_{1},\ldots ,\epsilon_{8}\right]}^{T}$, and $\Xi ≜{\left[\xi_{1}^{T},\ldots ,\xi_{8}^{T}\right]}^{T}$. This equation presents a unified representation of multi-source errors within the 24-channel measurement space. It is worth noting that Equation (9) serves as a forward generative error model. Rather than directly inverting this complete 24-dimensional physical parameterization, the proposed calibration algorithm employs a hierarchical, reduced-order identification strategy to enhance both identifiability and numerical robustness.

## 3. Proposed In Situ Calibration Framework

### 3.1. Construction of Randomized Constant-Magnitude Fields via 3D Helmholtz Coils

To break the bottleneck of traditional mechanical rotation calibration, the core of in situ scalar calibration lies in constructing an active excitation magnetic field that achieves fully omnidirectional spatial coverage and provides an absolute magnitude reference, all without requiring any physical rotation. To this end, this paper proposes utilizing a 3D Helmholtz coil to generate a magnetic field sequence with strictly constant magnitude and randomly traversing directions within the highly uniform central zone where the cubic Magnetic Gradient Tensor Array is located. The omnidirectional excitation capability of the proposed system originates from the structural configuration and control strategy of the 3D Helmholtz coil assembly. Specifically, the platform consists of three mutually orthogonal Helmholtz coil pairs, each of which predominantly generates one magnetic-field component along its corresponding physical axis. Owing to the linear superposition principle of magnetic fields, the resultant field at the geometric center can be synthesized as the vector sum of the three axis-wise coil outputs. Therefore, by continuously adjusting the three driving currents, the system is able to generate magnetic-field vectors with arbitrary orientations in three-dimensional space, rather than being restricted to a single rotation plane or a limited set of discrete attitudes. Moreover, the cubic MGT array is positioned within the high-uniformity central region of the coil assembly, where the residual spatial gradient of the excitation field is sufficiently small. Under this condition, all sensors approximately experience the same instantaneous magnetic-field vector, which provides the physical basis for using the generated field as a common omnidirectional calibration input. The implementation of this active excitation mechanism relies on two critical steps. First, the precise mapping relationship between the coil driving currents and the central magnetic field vector is established, accompanied by system-level inter-axis coupling compensation. Second, when combined with a highly stable closed-loop control strategy, a randomized directional magnetic field sequence strictly satisfying the spherical constraint (${∥B∥}^{2}=C$) is generated. This provides comprehensive and sufficient excitation conditions for the robust multi-parameter convergence of the 24-dimensional high-order error model formulated in the previous section.

#### Spatially Uniform Excitation and Active Background Field Cancellation

**Figure 2:**
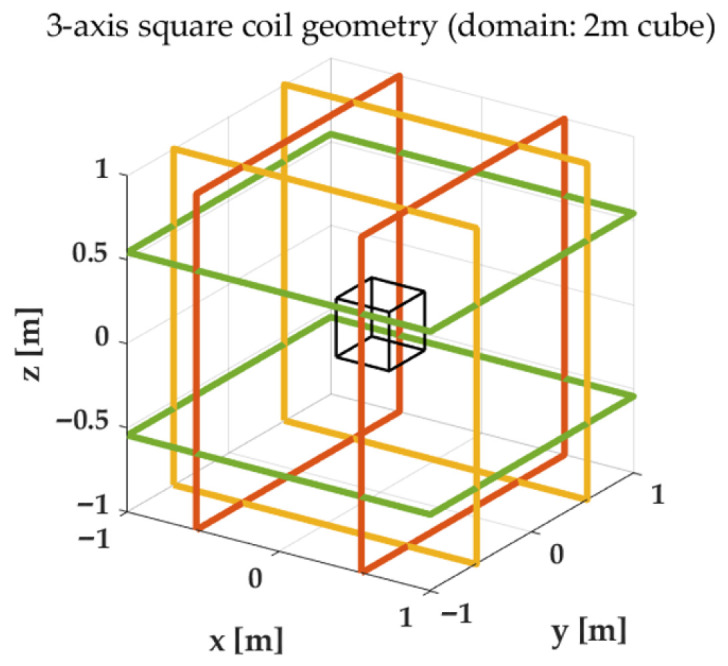
Helmholtz coil geometry and the uniform-field central region used for in situ calibration and background-field cancellation.

As illustrated in the hardware schematic in Figure 2 the 3D Helmholtz coil generator comprises three pairs of mutually orthogonal coaxial loop coils, which are responsible for independently exciting the magnetic field components along three physical orthogonal axes (denoted as the coil coordinate system, $O-X_{c}Y_{c}Z_{c}$).

According to the Biot–Savart law, a Helmholtz coil generates a highly uniform magnetic field with a minimal spatial gradient within a spherical volume near its geometric center. In the proposed in situ calibration architecture, the cubic Magnetic Gradient Tensor Array is positioned within this uniform central zone. This spatial configuration guarantees a key prerequisite for in situ calibration: under the ideal assumption that the residual spatial gradient is negligible, all spatially distributed sensors in the array observe the same true excitation magnetic field vector at any instant (i.e., $B_{1}^{A}=B_{2}^{A}=\cdots =B_{8}^{A}$).

However, in practical in situ experimental environments, the omnipresence of the geomagnetic field and slowly varying external magnetic disturbances inevitably superimpose an unknown ambient bias $B_{env}$ onto the measurement space. If uncompensated, directly superimposing the calibration magnetic field would result in a distorted magnetic vector with an unknown offset, fundamentally violating the strict spherical constraint (${∥B∥}^{2}=C$) required by the subsequent scalar calibration algorithm. It should be noted that the proposed in situ calibration framework is designed for practical laboratory environments rather than perfectly shielded magnetic conditions. In such environments, $B_{env}$ may include not only the geomagnetic background but also quasi-static disturbances from nearby electrical infrastructure and surrounding equipment. Accordingly, the purpose of the background cancellation step is to minimize the dominant environmental bias at the array center before the calibration sequence is applied.

In the proposed platform, a high-fidelity triaxial fluxgate magnetometer (Model CH-370, integrated into the coil system) is employed as the reference sensor. As detailed in Table 1, the performance specifications of the CH-370 magnetometer are significantly superior to those of the uncalibrated discrete array sensors, particularly exhibiting an ultra-low zero-field offset error (±5 nT) and a minimal three-dimensional orthogonality error (<0.1°).

Before generating the randomized constant-magnitude excitation sequence, the ambient magnetic field at the array center was first measured under zero-current conditions using the high-accuracy reference triaxial fluxgate sensor integrated into the Helmholtz coil system. Based on this real-time measurement, the control unit drove the tri-axial Helmholtz coils to generate an oppositely directed static compensation field ($-B_{env}$), thereby minimizing the residual background field in the central calibration region. After this initial cancellation step, the programmed excitation field was generated on top of the compensated background. In addition, during the calibration sequence, the same reference sensor continuously monitored the central field, and a PI-based feedback loop was used to compensate for low-frequency disturbances, coil-heating-induced drift, and slow ambient variations, so that the synthesized field magnitude remained as stable as possible throughout the experiment.

### 3.2. Current-Magnetic Field Coupling Modeling and Closed-Loop Sequence Generation

After active cancellation of the ambient background field, a tri-axial Helmholtz-coil assembly is employed to synthesize the excitation field required for in situ array calibration. In practical implementations, machining tolerances, slight non-orthogonal coil mounting, and electromagnetic mutual coupling among windings invalidate the ideal decoupled assumption that a single-axis current produces a purely single-axis field. To account for these nonidealities, we establish a linear current-to-field coupling model at the array center:

(10) $$B\left(k\right)=K\;I\left(k\right)+B_{0},$$

where $I\left(k\right)={\left[I_{x},\;I_{y},\;I_{z}\right]}^{T}$ is the commanded coil current vector, $B\left(k\right)={\left[B_{x},\;B_{y},\;B_{z}\right]}^{T}$ is the realized magnetic field at the center, $B_{0}$ denotes the residual bias after cancellation, and $K\in R^{3\times 3}$ is the coupling matrix. The diagonal entries of K represent the principal conversion gains, whereas the off-diagonal terms quantify cross-axis crosstalk.

The coupling matrix K is identified using a reference magnetometer placed at the array center. Specifically, *M* current vectors ${\left\{I^{\left(m\right)}\right\}}_{m=1}^{M}$ that sufficiently excite all three axes are applied, and the corresponding field responses ${\left\{B^{\left(m\right)}\right\}}_{m=1}^{M}$ are recorded synchronously. The residual bias $B_{0}$ is estimated from zero-current samples, and incremental observations are formed as $\Delta B^{\left(m\right)}=B^{\left(m\right)}-B_{0}$. Stacking $\Delta B^{\left(m\right)}$ and $I^{\left(m\right)}$ column-wise into ΔB and I, the least-squares estimate is obtained by

(11) $$\hat{K}=\Delta B\;I^{T}{\left(II^{T}\right)}^{-1},$$

where $II^{T}$ is required to be full rank. With $\hat{K}$ available, an arbitrary target field $B_{d}\left(k\right)$ can be mapped into the open-loop current command via the Moore–Penrose pseudo-inverse

(12) $$I_{d}\left(k\right)={\hat{K}}^{†}\left(B_{d}\left(k\right)-B_{0}\right).$$

To meet the scalar-calibration requirement of constant field magnitude and broad directional coverage, we set the desired magnitude to $H_{m}$ and generate isotropically distributed directions via Gaussian normalization:

(13) $$v\left(k\right)∼N\left(0,I_{3\times 3}\right),\;u\left(k\right)=\frac{v(k)}{∥v(k)∥},\;B_{d}\left(k\right)=H_{m}\;u\left(k\right).$$

Prior to actuation, practical constraints—including current limiting, slew-rate limiting, and low-pass smoothing—are applied to ensure compatibility with the power amplifier bandwidth and to suppress transient overshoot.

To mitigate magnitude drift induced by coil heating, slow gain variations, and low-frequency disturbances, we further introduce a feedback-based amplitude stabilization loop using the same reference magnetometer. Let $B_{m}\left(k\right)$ be the measured center field and $e\left(k\right)=B_{d}\left(k\right)-B_{m}\left(k\right)$ the tracking error. A PI correction is applied in the field domain and mapped back to currents through ${\hat{K}}^{†}$, yielding the final command:

(14) $$I\left(k\right)=I_{d}\left(k\right)+{\hat{K}}^{†}\left(K_{p}\;e\left(k\right)+K_{i}\sum_{\tau \leq k}e\left(\tau \right)\right),$$

where $K_{p}$ and $K_{i}$ are diagonal gain matrices. The resulting excitation sequence maintains a near-constant magnitude while exploring directions over $S^{2}$, thereby providing a high-quality in situ dataset for estimating the 24-channel array error parameters in subsequent calibration.

### 3.3. Hierarchical Calibration Algorithm

This section presents a hierarchical in situ calibration strategy for the cubic MGT array under constant-magnitude randomized excitation generated by the tri-axial Helmholtz coil system. The array remains stationary throughout the procedure, and no mechanical rotation or external attitude reference is required. It is worth emphasizing that the full model in Section 2 serves as a physics-based generative description of multi-source error propagation, whereas the calibration strategy developed here is an identifiable reduced-order inversion derived under the near-uniform excitation condition at the coil center. Because all sensors ideally observe the same latent magnetic field vector at each instant in this region, the original 24-channel error model can be reformulated as a set of sensor-specific affine observations of a common hidden input. Under this condition, directly estimating all physical parameters in a single scalar-only optimization becomes poorly conditioned. Therefore, instead of pursuing one-to-one recovery of every physical error source, we factorize the inverse calibration into two stages: relative inter-sensor consistency alignment and common absolute scalar normalization. This factorized formulation improves identifiability and numerical stability while preserving the practical correction capability required for array-wide tensor reconstruction.

#### 3.3.1. Array-Level Misalignment Error

Within the coil’s near-uniform region, all sensors should observe the same instantaneous magnetic field vector in the ideal case; thus, discrepancies among channels primarily originate from sensor-to-sensor response mismatch (scale, non-orthogonality, bias) and installation deviations. We select sensor 1 as the reference and denote its measurement sequence by $B_{\mathrm{ref}}=B_{1}$. For each peripheral sensor n=2,…,8, an affine alignment model is introduced:

(15) $$B_{\mathrm{ref}}\approx B_{n}T_{n}+t_{n}^{T},$$

where $T_{n}\in R^{3\times 3}$ and $t_{n}\in R^{3}$ represent a linear mapping and an offset, respectively. The parameters $\left(T_{n},t_{n}\right)$ are estimated by solving:

(16) $$\left(T_{n},t_{n}\right)=\arg\min_{T,t}{∥B_{\mathrm{ref}}-B_{n}T-t^{T}∥}_{F}^{2}+\lambda {∥T∥}_{F}^{2},$$

where λ is a small regularization parameter and ${∥\cdot ∥}_{F}$ denotes the Frobenius norm. In principle, this fitting problem is well posed when the excitation samples provide sufficiently rich three-dimensional coverage. In the proposed calibration framework, the tri-axial Helmholtz coil system generates a constant-magnitude magnetic-field sequence with randomized orientations distributed over the unit sphere, so that the acquired samples are highly overdetermined and span the three-dimensional excitation space effectively. This omnidirectional excitation significantly improves the identifiability of the affine alignment model. Nevertheless, to further enhance numerical robustness against measurement noise, imperfect directional coverage, and possible near-degeneracy of the regression matrix, we adopt a Tikhonov-regularized least-squares formulation.

The aligned output is then defined as

(17) $$Y_{n}=B_{n}T_{n}+t_{n}^{T},\;n=2,\ldots ,8,$$

with $Y_{1}=B_{\mathrm{ref}}$. This step enforces spatial consistency across the array by mapping all measurement channels into a common reference coordinate frame. Crucially, it should be interpreted as estimating an equivalent transformation to compensate for array-level affine inconsistencies, rather than explicitly isolating each physical error component. By absorbing these dominant geometric mismatches during the alignment stage, the subsequent scalar calibration is effectively decoupled, allowing it to focus strictly on normalizing sensor-level intrinsic errors.

#### 3.3.2. Constant-Magnitude Calibration on the Reference Sensor

After consistency alignment, the array still lacks a unified absolute scale and bias level. Using the constant-magnitude randomized excitation with target magnitude $H_{m}$, we calibrate the reference channel by estimating a global correction matrix $A^{-1}\in R^{3\times 3}$ and bias $b\in R^{3}$ such that the corrected reference output satisfies the scalar constraint. Here, $A^{-1}$ is a shared global correction matrix applied after the array-level affine alignment, rather than a sensor-specific transformation. Its role is to map the aligned measurements into a common calibrated field space satisfying the constant-magnitude constraint. To ensure a physically meaningful three-dimensional mapping, $A^{-1}$ is required to be nonsingular, and a symmetry constraint is imposed to remove the rotational ambiguity inherent in scalar-only calibration. The corrected reference field is written as

(18) $$H_{1}=\left(Y_{1}-b^{⊤}\right)A^{-1},$$

and the constant-magnitude requirement is enforced by

(19) $$∥H_{1}{\left(k,:\right)∥}_{2}\approx H_{m},\;k=1,\ldots ,N.$$

Accordingly, we solve

(20) $$\min_{A^{-1},b}\sum_{k=1}^{N}{\left(∥\left(Y_{1}\left(k,:\right)-b^{⊤}\right)A^{-1}{∥}_{2}^{2}-H_{m}^{2}\right)}^{2},$$

subject to a symmetry constraint on $A^{-1}$, which reduces the effective degrees of freedom, improves identifiability, and enhances numerical robustness in the scalar-calibration stage.

#### 3.3.3. Parameter Composition and Array-Wide Correction

Finally, the alignment parameters $\left\{\left(T_{n},t_{n}\right)\right\}$ and the scalar-calibration parameters $\left(A^{-1},b\right)$ are composed to produce a unified correction for all sensors. For the reference sensor,

(21) $$H_{1}=\left(Y_{1}-b^{⊤}\right)A^{-1},$$

and for each peripheral sensor,

(22) $$H_{n}=\left(Y_{n}-b^{⊤}\right)A^{-1}=\left(B_{n}T_{n}+t_{n}^{⊤}-b^{⊤}\right)A^{-1},\;n=2,\ldots ,8.$$

In this composition, the affine alignment parameters $T_{n}$ first map each peripheral sensor output into the common reference frame, while the shared correction $\left(A^{-1},b\right)$ then performs a common absolute normalization in the aligned frame. The matrix $T_{n}$ is estimated using a Tikhonov-regularized least-squares scheme based on an overdetermined omnidirectional calibration dataset, which improves numerical conditioning and enhances robustness against noise and possible near-degeneracy of the regression problem. Therefore, the final correction can be interpreted as the composition of a numerically stable inter-sensor consistency alignment and a shared scalar normalization.

#### 3.3.4. Overall Execution Procedure of the Proposed Framework

For clarity, the complete execution procedure of the proposed rotation-free scalar calibration framework is summarized in Algorithm 1.
**Algorithm 1:** Execution Procedure of the Proposed Rotation-Free Scalar Calibration Framework
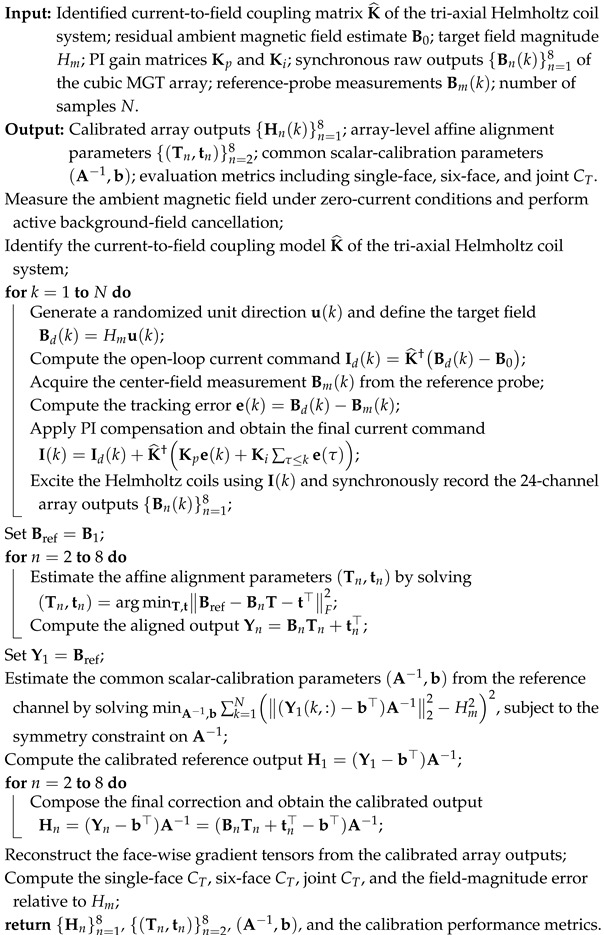


## 4. Experimental Validation

### 4.1. Experimental Configuration and Verification Framework

**Figure 3:**
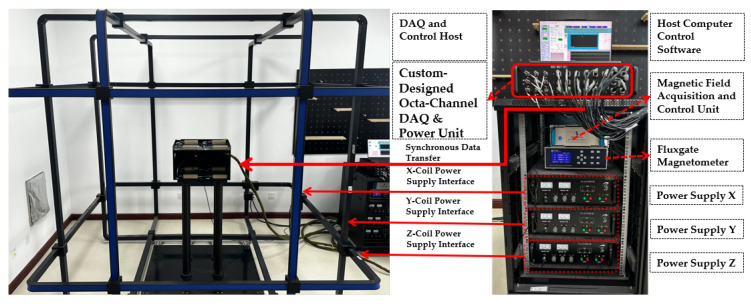
Hardware configuration of the experimental platform for in situ calibration, featuring the 3D Helmholtz coil uniform-field generator (**left**) and the integrated data acquisition and control system (**right**).

To comprehensively evaluate the effectiveness and reliability of the proposed in situ rapid calibration method for the cubic Magnetic Gradient Tensor Array, we established a dedicated experimental platform, as shown in Figure 3.

The platform is built around a three-dimensional (3D) Helmholtz coil system composed of three mutually orthogonal coil pairs. Each pair consists of two identical coaxial coils separated by one coil radius, thereby generating a highly uniform magnetic field near the geometric center. The array channels are implemented with Mag690-FL100 fluxgate magnetometers, whose key performance parameters are summarized in Table 2.

The experimental procedure followed a “background cancellation–constant-magnitude excitation–synchronous acquisition” pipeline. The experiments were conducted in a normal laboratory environment rather than in a fully magnetically shielded room. Therefore, special care was taken to mitigate EMI/EMC effects during data acquisition. First, the ambient field was measured under zero-current conditions and actively compensated so that the residual center bias became negligible. Specifically, before each calibration run, the ambient magnetic field at the array center was measured using the reference triaxial fluxgate sensor, and the tri-axial Helmholtz coil system generated an oppositely directed compensation field to suppress the dominant environmental bias in the central calibration region. Next, the host computer issued a predesigned 3D excitation sequence with constant magnitude and randomized orientations. The control unit converted the target field $B_{d}\left(k\right)$ into tri-axial current commands using the identified current-to-field coupling model, while synchronously recording the 24-channel array outputs and the reference measurements. To ensure precise dynamic regulation of the excitation field, a closed-loop stabilization mechanism was used: target commands were sent to the control unit via Ethernet, while real-time field measurements from the reference probe were fed back through RS232; a proportional-integral (PI) controller compensated current drift, coil-heating-induced gain variations, and low-frequency environmental disturbances. As a result, the synthesized excitation satisfies $∥B\left(k\right)∥\approx H_{m}$ throughout the sequence while maintaining broad directional coverage over $S^{2}$. To further reduce interference from surrounding electrical infrastructure and routine laboratory activity, the experiments were preferentially carried out during relatively quiet nighttime periods, when intermittent magnetic disturbances from nearby large-power equipment were less pronounced. Therefore, the measurement environment should be regarded as magnetically quiet, but not ideally interference-free. Finally, we quantified calibration performance by comparing the single-face $C_{T}$, six-face $C_{T}$, and field-magnitude error relative to $H_{m}$ before and after calibration.

### 4.2. Array-Level Performance Synthesis: Consistency Evaluation of Magnetic Gradient Invariants

In this central uniform region, the ideal spatial magnetic gradient satisfies ∇B=0. To quantitatively evaluate measurement accuracy and the suppression of residual errors after calibration, the Frobenius norm of the MGT is introduced as a scalar invariant, denoted by $C_{T}$:

(23) $$C_{T}={∥G∥}_{F}=\sqrt{\sum_{i,j\in \{x,y,z\}}G_{ij}^{2}}$$

where $G_{ij}$ represents the individual components of the estimated gradient tensor matrix G. The magnitude of $C_{T}$ directly indicates the total deviation from the ideal zero-gradient state, effectively capturing the combined effects of sensor-level stochastic noise and systematic bias.

**Figure 4:**
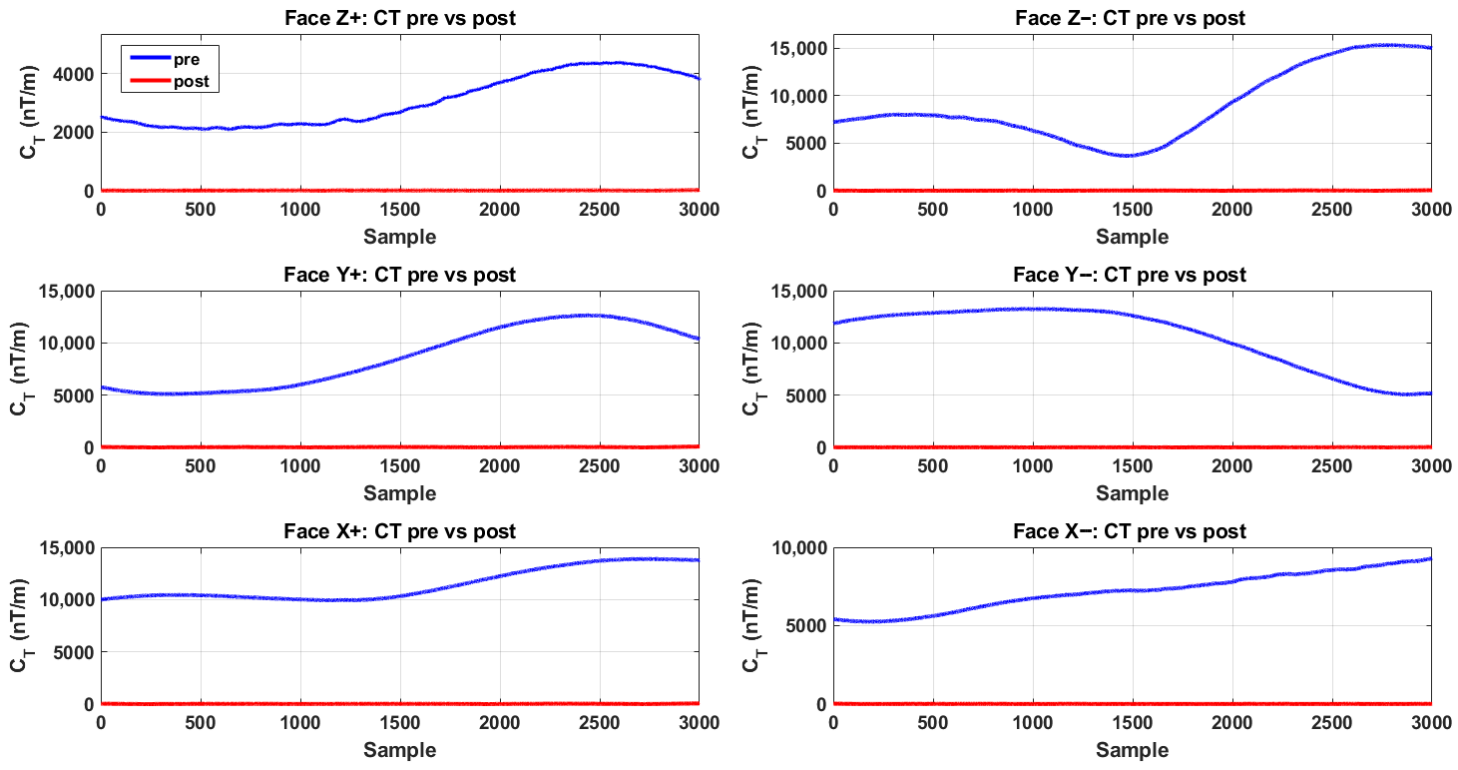
Face-wise $C_{T}$ time-series comparison on the six cubic faces before and after in situ calibration.

Figure 4 compares the face-wise $C_{T}$ time series reconstructed on the six cubic faces (±z, ±y, ±x) before and after applying the proposed in situ calibration. Prior to calibration, all faces exhibit pronounced nonzero $C_{T}$ baselines and slow-varying drifts, with amplitudes persistently in the $10^{3}$–$10^{4}$ nT/m range, indicating that even under nominally uniform excitation, inter-sensor mismatch and residual bias are strongly amplified by the finite-difference tensor reconstruction. After calibration, the traces on every face collapse to a near-zero baseline, and the residual fluctuations remain at the single-/tens-of-nT/m level over the entire record, demonstrating a consistent suppression of face-wise reconstruction error.

**Figure 5:**
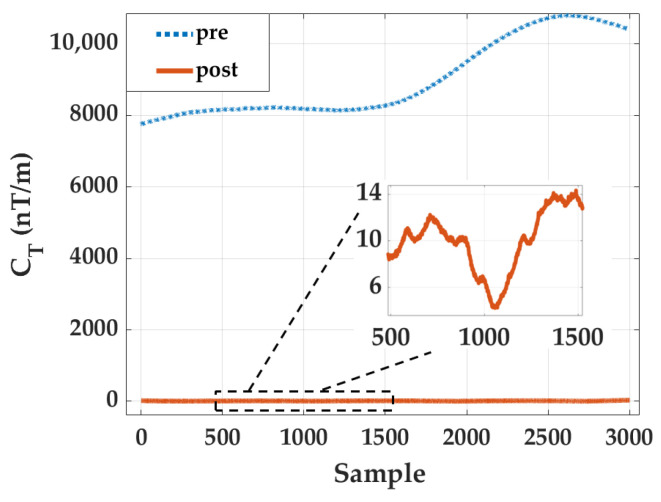
Joint $C_{T}$ time-series comparison of the six-face aggregated invariant before and after in situ calibration, with an inset of post-calibration residual fluctuations.

As summarized in Table 3, the face-wise statistical metrics further corroborate the effectiveness of the proposed calibration. Across the six faces, the RMS of $C_{T}$ decreases from $3.17\times 10^{3}$–$1.15\times 10^{4}$ nT/m to 6.03–17.21 nT/m, corresponding to reduction factors of $5.3\times 10^{2}$–$1.5\times 10^{3}$. Likewise, the mean decreases from $3.06\times 10^{3}$–$1.14\times 10^{4}$ nT/m to 5.74–15.17 nT/m, with reduction factors of $5.3\times 10^{2}$–$1.6\times 10^{3}$. Meanwhile, the variance drops from $7.36\times 10^{5}$–$1.42\times 10^{7}$ to 3.40–65.89 nT^2^/m^2^. The comparable gains observed across all six measurement planes indicate that the calibration does not merely improve a subset of channels; rather, it enforces array-wide consistency that is directly reflected in each face’s tensor reconstruction. Building on these face-wise results, we further quantify the overall array-level improvement using the joint $C_{T}$ index aggregated over all faces, as shown in Figure 5 and Table 4.

Figure 5 presents the time-series comparison of the joint $C_{T}$ before and after applying the proposed in situ calibration, where the solid blue curve denotes the pre-calibration result and the dotted magenta curve denotes the post-calibration result. Prior to calibration, the aggregated invariant exhibits a large, nonzero baseline with slow temporal drift, reaching values on the order of $10^{4}$ nT/m. This behavior reflects the cumulative amplification of inter-sensor mismatch and systematic bias when the gradient tensor is reconstructed from multiple finite-difference planes. In contrast, after calibration, the joint $C_{T}$ collapses to a near-zero baseline across the entire sequence. The inset further illustrates that the residual fluctuations are confined to the 6 to 14 nT/m level, demonstrating that the array-wide distortion has been effectively removed.

The statistical indicators summarized in Table 4 further confirm this substantial improvement. The RMS of the joint $C_{T}$ decreases from 9074.65 nT/m to 11.51 nT/m, corresponding to a reduction of 99.87%. Similarly, the mean value drops from 9010.92 nT/m to 10.51 nT/m, while the variance is reduced from $1.15\times 10^{6}$ to 22.03 (nT/m)^2^, representing a reduction of 99.998%. Such multi-order-of-magnitude suppression of the joint invariant demonstrates that the proposed calibration method effectively restores the theoretical zero-gradient condition expected in a uniform field environment. More importantly, the consistency between the face-wise improvements and the aggregated invariant indicates that the calibration operates coherently across all 24 measurement channels, rather than compensating individual sensors independently.

**Figure 6:**
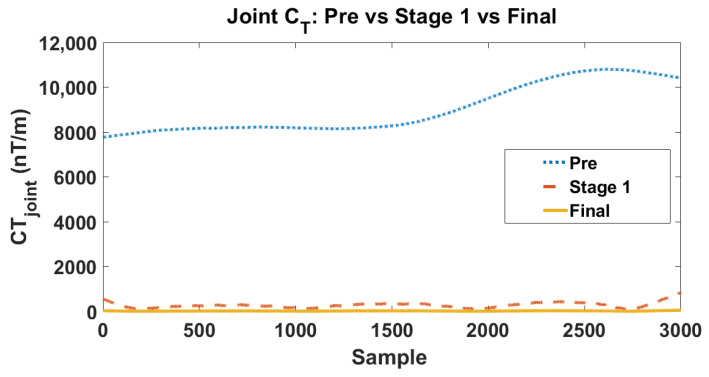
Evolution of the joint tensor invariant ($C_{T}$) across three critical intermediate states: uncalibrated (Pre), post-affine alignment (Stage 1), and fully calibrated (Final).

To further clarify the respective roles of the two stages in the proposed hierarchical calibration framework, we separately evaluate the intermediate result after Stage 1 and the final result after Stage 2. The corresponding joint $C_{T}$ evolutions are shown in Figure 6. As illustrated in Figure 6, the uncalibrated array (blue dotted line) exhibits severe pseudo-gradient fluctuations, with the joint $C_{T}$ surging into the 8000–11,000 nT/m range, yielding an initial root-mean-square (RMS) of 9074.65 nT/m. This intuitively confirms the theoretical premise that even minute inter-sensor inconsistencies are strongly amplified during finite-difference tensor reconstruction. Following the execution of Stage 1, the joint $C_{T}$ trajectory (orange dashed line) undergoes a precipitous drop, decreasing from the $10^{4}$ nT/m level to the $10^{2}$ nT/m level. This massive compression—reducing the error by nearly two orders of magnitude—explicitly demonstrates that the dominant source of tensor distortion stems from array-level affine mismatch. By mapping the spatially distributed sensor outputs into a unified reference frame, Stage 1 effectively neutralizes this primary error source, fundamentally restoring the relative spatial consistency across the 24 measurement channels. Despite the substantial improvement achieved in Stage 1, a residual baseline error of several hundred nT/m persists, indicating that relative spatial alignment alone is insufficient to entirely recover the true field gradients. The subsequent implementation of Stage 2—leveraging the robust constant-magnitude constraint—further compresses these residuals, collapsing the joint $C_{T}$ trajectory (yellow solid line) to a near-zero baseline with an ultimate RMS of 11.51 nT/m. Compared to the pre-calibration state, the full hierarchical framework achieves a staggering error suppression ratio (a 99.87% reduction). Although the absolute numerical reduction in this second phase is smaller than that of Stage 1, it is physically indispensable. It functions as a definitive precision refinement, successfully eliminating the residual absolute scale and zero-bias mismatches that inherently evade relative affine alignment. Collectively, this stage-wise evolution rigorously validates the rationale behind the proposed hierarchical design. Stage 1 serves as the primary engine for restoring inter-sensor spatial consistency, suppressing the bulk of the pseudo-gradient artifacts; meanwhile, Stage 2 acts as the crucial optimizer for absolute scalar fidelity. This decoupled optimization strategy not only effectively averts the mathematical ill-conditioning typical of high-dimensional global optimization but also aligns perfectly with the physical hierarchy of multi-source errors in complex MGT arrays.

### 4.3. Individual Sensor Linearity and Scale Consistency Evaluation

While the joint $C_{T}$ index validates the collective consistency of the array, a rigorous assessment of calibration efficacy also requires evaluating the response fidelity of each sensing unit. This ensures that global consistency originates from the high-fidelity linearized output of each constituent magnetometer rather than from a purely mathematical optimization of invariants. To this end, the relative magnitude error ($E_{\mathrm{rel}}$) is introduced to quantify the linearization and scale consistency of each sensor:

(24) $$E_{\mathrm{rel}}=\frac{\left|∥B_{i,\mathrm{final}}∥-H_{m}\right|}{H_{m}}\times 100\%.$$

where $B_{i,\mathrm{final}}$ denotes the calibrated magnetic-field magnitude measured by the *i*-th sensor.

**Figure 7:**
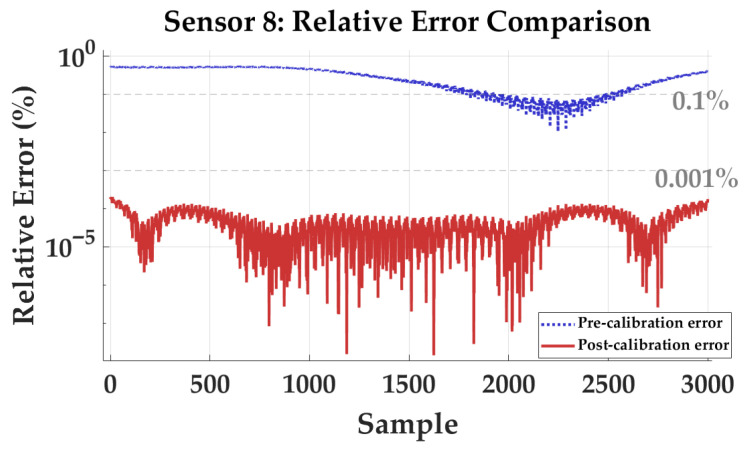
Semi-logarithmic comparison of the relative magnitude error for Sensor 8 before and after in situ calibration under randomized constant-magnitude excitation.

Figure 7 illustrates the comparison of relative magnitude errors for Sensor 8 before and after calibration under random constant-magnitude excitation, shown on a semi-logarithmic scale. Prior to calibration, the relative error fluctuates between $10^{-1}\%$ and $10^{0}\%$, with a mean value of 0.2978%. After applying the proposed method, the error trajectory is suppressed to the $10^{-5}\%$ level, and the post-calibration mean decreases to 0.000047%.

Detailed statistics for the full eight-sensor array are summarized in Table 5. Before calibration, the mean errors are non-uniformly distributed from 0.2978% to 2.6453%, revealing significant intrinsic scaling discrepancies among sensors. After calibration, the RMS errors of all sensors are consistently reduced below 0.0001%. The array-wide average mean error decreases from 1.8275% to 0.000036%, corresponding to an improvement of approximately five orders of magnitude.

The transition from percent-level deviations to a residual floor at the $10^{-5}\%$ level is attributed to robust identification of sensor-level parameters in the in situ framework. The pronounced pre-calibration errors stem from coupled sensitivity mismatches (scale factors) and non-orthogonality within each triaxial magnetometer, which cause reconstructed field magnitudes to deviate from the true excitation as orientation changes. The high post-calibration uniformity indicates that the algorithm effectively normalizes scale factors and decouples cross-axis sensitivities in a unified array coordinate frame. Furthermore, the convergence of RMS values to the $10^{-5}\%$ level suggests that nonlinearities and residual biases of individual sensors are effectively mitigated. This high-precision linearization at the unit-sensor level is a prerequisite for accurate spatial differencing, ensuring that gradient tensor components are dominated by true field variations rather than sensor-induced artifacts.

### 4.4. Comparison with Existing Methods

To further validate the effectiveness of the proposed in situ calibration framework, a comparative analysis was conducted against the conventional rotation-based calibration method reported in [15]. In particular, the fast calibration algorithm proposed in [15] was applied to the same experimental dataset acquired under identical Helmholtz-coil excitation conditions. The calibrated sensor outputs were then used to reconstruct the corresponding joint $C_{T}$ sequences for performance comparison.

It is worth noting that conventional rotation-based calibration methods typically require physically rotating the magnetometer array within a uniform magnetic field to generate calibration samples. However, for multi-sensor magnetic gradient tensor arrays, such mechanical rotations inevitably introduce additional uncertainties. Small rotation inaccuracies, axis misalignments, and mechanical vibration can perturb the relative geometry among sensors, thereby introducing extra orientation errors and scale inconsistencies that propagate into the tensor reconstruction process. These effects are particularly critical for gradient-based measurements, where even small sensor misalignments may produce significant pseudo-gradient artifacts. In contrast, the proposed calibration method operates entirely in situ, without requiring any physical rotation of the sensor array. Instead, a three-dimensional constant-magnitude magnetic excitation sequence is generated electronically by the Helmholtz coil system, thereby allowing the calibration process to be performed while preserving the intrinsic array geometry. This design effectively eliminates rotation-induced errors and ensures that the calibration parameters accurately reflect the true operational configuration of the array.

**Figure 8:**
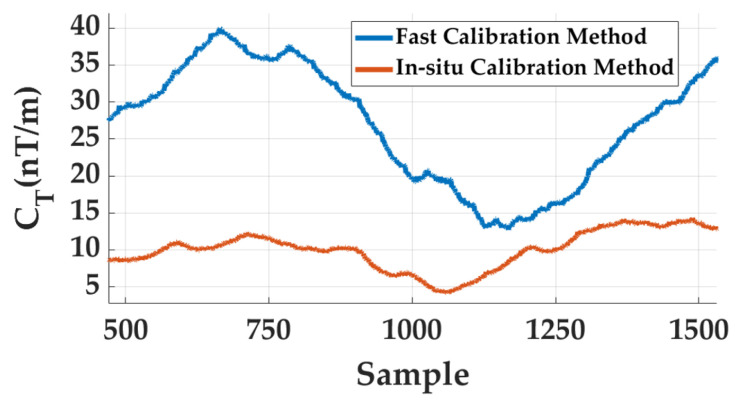
Comparison of the joint $C_{T}$ time series obtained by the conventional rotation-based fast calibration method and the proposed in situ calibration method under identical Helmholtz-coil excitation conditions.

The comparison results are illustrated in Figure 8. It can be observed that although the conventional fast calibration method reduces part of the gradient distortion, the resulting joint $C_{T}$ level remains noticeably higher and exhibits larger temporal fluctuations. By comparison, the proposed in situ calibration method consistently maintains a significantly lower joint $C_{T}$ baseline throughout the measurement sequence, indicating more effective suppression of pseudo-gradient components caused by multi-channel scale mismatch and bias inconsistencies.

Quantitative statistics further confirm this improvement. As summarized in Table 6, the mean value of the joint $C_{T}$ decreases from 26.66 nT/m using the conventional fast calibration method to 9.95 nT/m with the proposed in situ calibration approach. Meanwhile, the RMS reduces from 27.90 nT/m to 10.29 nT/m, and the variance decreases from 67.38 to 6.89, demonstrating a substantial reduction in both the magnitude and fluctuation of the reconstructed tensor invariant. These results indicate that the proposed in situ calibration strategy not only avoids the intrinsic limitations of rotation-based calibration but also provides more robust correction of multi-sensor inconsistencies within the array. Consequently, it delivers more stable and reliable magnetic gradient tensor measurements, which are essential for high-precision magnetic anomaly detection and target localization.

## 5. Conclusions

High-precision calibration of spatially distributed magnetic gradient tensor arrays has long been hindered by the rotational dynamic errors and mechanical tolerances inherent in traditional methods. This paper proposes a completely rotation-free, in situ scalar calibration framework based on a cubic Magnetic Gradient Tensor Array. By integrating active background field cancellation, randomized constant-magnitude excitation, and a hierarchical decoupled optimization algorithm, the proposed method achieves global parameter convergence across the 24-dimensional measurement space without altering the array’s operational pose.

The experimental results show that the proposed framework markedly improves array consistency under the tested uniform-field in situ calibration condition. At the array level, the joint tensor invariant $C_{T}$ is significantly suppressed in both magnitude and temporal fluctuation, indicating effective reduction of pseudo-gradient artifacts introduced by multi-channel mismatch. At the sensor level, the field-magnitude residual relative to the prescribed constant-magnitude excitation is also substantially reduced after calibration, showing that the aligned array outputs are more closely normalized to a common scale and bias reference. Finally, benchmarking against conventional fast calibration reveals that by replacing dynamic mechanical rotations with our stationary closed-loop excitation, the proposed method simultaneously eradicates motion-induced pseudo-gradients and compensates for environmental background drifts, thereby substantially enhancing both absolute accuracy and stochastic stability.

Overall, the proposed rotation-free framework provides a practical calibration route for cubic MGT arrays operating without mechanical turntables. The present results support its effectiveness for improving array self-consistency and suppressing reconstruction artifacts in uniform-field calibration experiments, and they provide a useful basis for future validation in downstream tasks such as magnetic target localization and field deployment.

## Figures and Tables

**Table 1:** Parameter specifications of the reference magnetometer (CH-370).

Parameter	Specification Value
Measurement Range	±100 μT
Zero-field Offset Error	±5 nT
Resolution	0.01 nT
Accuracy	<0.2% ± 5 nT of the reading
Sensor Noise	$6\;{\mathrm{pT}}_{\mathrm{RMS}}/\sqrt{\mathrm{Hz}}$ at 1Hz
3D Orthogonality Error	<0.1°

**Table 2:** Specifications of the Mag690-FL100 magnetometer used in the experiments.

Accuracy Specification	Parameter Value
Background Noise (1 Hz)	≤25 ${\mathrm{pT}}_{\mathrm{RMS}}/\sqrt{\mathrm{Hz}}$
Linearity Error	0.01% F.S.
Orthogonality Error	<1°
Offset Error	±100 nT
Offset Temperature Drift	±2 nT/°C
Calibration Error	±1%

**Table 3:** Face-wise statistical indicators of $C_{T}$ before and after calibration.

Condition	Metric	$C_{T}$ Z+	$C_{T}$ Z−	$C_{T}$ Y+	$C_{T}$ Y−	$C_{T}$ X+	$C_{T}$ X−
Post-calibration	RMS (nT/m)	6.03	12.17	17.21	7.43	14.33	7.33
	Mean (nT/m)	5.74	10.62	15.17	6.53	12.77	6.94
	Variance ((nT/m)^2^)	3.40	35.22	65.89	12.60	42.20	5.52
Pre-calibration	RMS (nT/m)	3174.39	9640.84	9120.04	11,017.14	11,545.27	7295.77
	Mean (nT/m)	3056.25	8873.84	8651.76	10,631.77	11,445.85	7197.02
	Variance ((nT/m)^2^)	736,346.00	14,205,469.84	8,324,886.26	8,344,954.98	2,286,524.65	1,431,744.32

**Table 4:** Overall statistical indicators of the joint $C_{T}$ before and after calibration.

Metric	Pre-Calibration	Post-Calibration	Reduction (%)
RMS (nT/m)	9074.65	11.51	99.87
Variance ((nT/m)^2^)	1,153,019.36	22.03	99.998
Mean (nT/m)	9010.92	10.51	99.88

**Table 5:** Per-sensor percentage errors before and after calibration.

Sensor	Pre-Mean (%)	Post-Mean (%)	Pre-RMS (%)	Post-RMS (%)
Sensor 1	2.435304	0.000022	2.453922	0.000027
Sensor 2	1.917486	0.000028	1.929163	0.000034
Sensor 3	2.618505	0.000033	2.618858	0.000041
Sensor 4	1.567760	0.000054	1.583778	0.000067
Sensor 5	1.401455	0.000030	1.542734	0.000038
Sensor 6	2.645306	0.000025	2.669565	0.000035
Sensor 7	1.736717	0.000046	1.768370	0.000056
Sensor 8	0.297828	0.000047	0.348465	0.000059
Mean	1.827545	0.000036	1.864357	0.000045

**Table 6:** Statistical comparison of the joint $C_{T}$ index between the conventional fast calibration method and the proposed in situ calibration method.

Metric	Fast Calibration Method [15]	Proposed In Situ	Reduction (%)
Mean (nT/m)	26.66	9.95	62.68
Variance (nT^2^/m^2^)	67.38	6.89	89.77
RMS (nT/m)	27.90	10.29	63.12

## Data Availability

The original contributions presented in this study are included in the article material. Further inquiries can be directed to the corresponding author.

## Abbreviations

The following abbreviations are used in this manuscript:

MGT	Magnetic Gradient Tensor
$C_{T}$	Contraction Tensor
ARCS	Array Reference Coordinate System
UXO	Unexploded Ordnance
PI	Proportional–Integral Controller
RMS	Root Mean Square
LS	Least Squares
